# Dual-Energy Heart CT: Beyond Better Angiography—Review

**DOI:** 10.3390/jcm10215193

**Published:** 2021-11-07

**Authors:** Piotr Tarkowski, Elżbieta Czekajska-Chehab

**Affiliations:** Department of Radiology, Medical University of Lublin, ul. Jaczewskiego 8, 20-090 Lublin, Poland; czekajska@gazeta.pl

**Keywords:** dual-energy CT, heart, coronary arteries, spectral computed tomography

## Abstract

Heart CT has undergone substantial development from the use of calcium scores performed on electron beam CT to modern 256+-row CT scanners. The latest big step in its evolution was the invention of dual-energy scanners with much greater capabilities than just performing better ECG-gated angio-CT. In this review, we present the unique features of dual-energy CT in heart diagnostics.

## 1. Introduction

While cardiac CT was developed to assess coronary arteries and heart anatomy, thanks to technological development, we can evaluate much more than that alone. The first step in its development was to increase the number of detector rows and the temporal and spatial resolution of the scanners. The next important step was the introduction of a dual-source CT scanner by Siemens Healthcare in 2006, capable of working in the dual-energy mode [1]. Since then, different vendors have produced dual-energy (DECT) scanners of their own design, each with sets of unique advantages and disadvantages [2]. In this review, we summarize the possibilities of dual-energy CT scanners in heart diagnostics, and the limitations and advantages of different types of DECT scanners. 

## 2. Fundamentals of Dual-Energy CT

Conventional single-energy CT (SECT) scanners generate a beam of X-ray photons of different energies with maximal energy equal to the value of the peak voltage of the X-ray lamp (kVp)—a beam of that kind is polychromatic [3] and images represent the attenuation of photons of all energies in each voxel. 

Dual-energy CT scanners acquire two sets of data with different energy levels for each voxel and create two sets of images independently for each energy, similarly to SECT [2]. Photons that travel through a patient’s tissues interact with them through two main processes: Compton scattering and the photoelectric effect. In the photoelectric effect, the X-ray photon interacts with an atom’s K-shell electron, causing its ejection from the shell. The likelihood of such event is greatest if the energy of the X-ray photon is equal or slightly above the binding energy of electrons to the K-shell, which is different for each element. The binding energy increases proportionally to the atomic number. Compton scattering is the ejection of electrons from the outer shell of an atom, and it mainly occurs in elements with low atomic numbers such as hydrogen Z = 1, oxygen Z = 8 or carbon Z = 6 [2,3].

## 3. Diagnostic Capabilities of DECT

All types of dual-energy CT scanners, regardless of their technical concept, have similar capacities and do not have some of the single-energy CT limitations related to scanning with one polychromatic beam of X-ray. Moreover, DECT scanners have some unique functions that are not available in traditional devices.

### 3.1. Virtual Monoenergetic Images (VMIs)

DECT scanners can reconstruct images representing the attenuation of single-energy X-ray photons in each voxel, which are called virtual monoenergetic images (VMIs), while SECT images represent the attenuation of an entire spectrum of emitted photons [2]. The polychromatic nature of X-ray beams is a source of many limitations and artifacts of SECT, which are not present or are significantly reduced in VMIs. VMIs can be image- and projection-based depending on type of scanner [4]. Image-based VMIs are created by bending images obtained with different kV in different proportions in order to obtain VMIs with specific keV. Projection-based VMIs are created from maps of two substance concentrations. Most commonly used is the pairing of iodine and water, which is generated from raw data obtained from the same location of the X-ray lamp but with different kVp, and then VMIs are reconstructed from these maps [5]. Projection-based VMIs are more effective in reducing the beam-hardening artifact than image-based ones [4,6]. 

Depending on the energy level of the VMI, the same tissue has different densities and contrast enhancements. The lower-energy VMIs are more sensitive to iodine [7,8,9]. The closer the energy of the VMI to the K-edge value of iodine (K = 33.2 keV), the more sensitive to iodine the image is. This condition is true for every substance, which is a great opportunity to create new contrast materials [10,11]. If the K-edge value is significantly different for two substances, they can be virtually separated from each other, such as the case for iodine from carbon, oxygen or nitrogen—organic tissues. This is the principle of virtual noncontrast (VNC) imaging [7]. 

### 3.2. Material Specific Images and Virtual Noncontrast Images (VNC)

Each substance has its own unique profile of absorption of X-rays with specific energy. Using images obtained with two different energies, we can calculate the concentration of any substance with the known attenuation curve [2]. This is possible thanks to the photoelectric effect, which is Z-number dependent [3]. Due to this relation, DECT scanners can create images coded with concentrations of certain substances instead of X-ray attenuation in voxels; another way of using these data is to remove specific substances from an image, e.g., iodine or calcium. By removing iodine, we can obtain an image very similar to a noncontrast image—they are called virtual noncontrast (VNC) images [5,7,12]. It has been proved by several authors that VNC images can successfully replace noncontrast scans in the case of cardiac examinations and the examination of other anatomical regions [4,10,13,14]. However, this technology has some limitations and cannot completely remove the attenuation from highly concentrated contrasts, e.g., in the SVC and artifacts associated with it [15]. 

### 3.3. Effective Atomic Number Images

Having two sets of X-ray attenuation values for each voxel allows one to determine the composition of tissue by calculating the effective atomic number (Z_effective_). This is the average atomic number of all atoms in the voxel. These values can be displayed as a grayscale image or as color overlay on top of standard image, or as a VMI [13]. The effective Z number image can be used to differentiate two highly hyperdense structures, e.g., iodine in the lumen of the artery and calcification in its walls [4]. 

## 4. Types of DECT Scanners from Different Manufacturers

**Figure 1:**
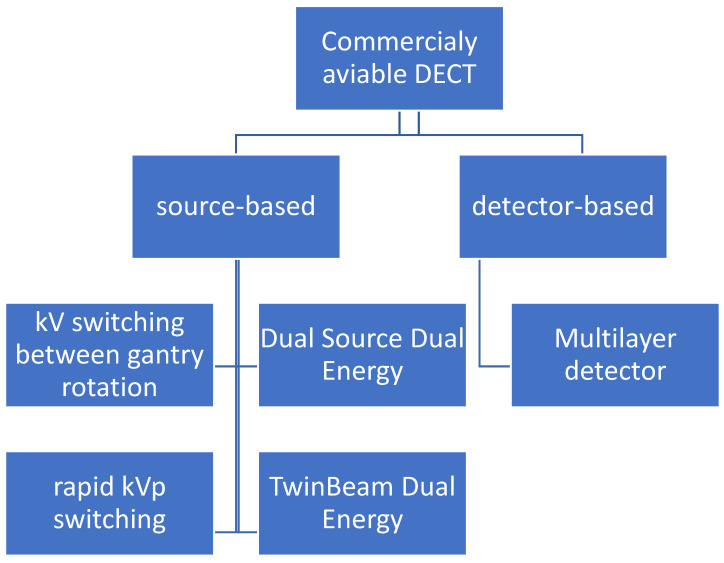
Technical source of dual-energy data. There are five commercially available types of CT scanners: four of them are source-based and only one is detector-based. DECT, dual-energy CT.

There are two types of dual-energy CT scanners: source-based and detector-based (Figure 1) [16]. 

More commonly used are source-based CT scanners: dual-source, twin-beam, rapid kVp switching and sequential kVp switching. The only operational detector-based scanner is the layer detector, designed by Philips Healthcare, Best, Netherlands. The ideal detector of spectral imaging—the photon counting detector—has been engineered for many years, but it is still not suitable to be used in CT scanners. Small photon counting detectors have been successfully used in mammography. Each of these technologies has its own advantages and disadvantages, which is discussed in more detail (Table 1).

### 4.1. Dual-Source Dual-Energy CT

Siemens-designed dual-source CT scanners comprise two sets of X-ray lamps and detectors shifted relative to each other by 90 or 95 degrees (Table 2). These scanners can operate as dual-energy ones when each lamp is powered with a different kVp [2]. These scanners create monoenergetic images by blending images obtained with two detectors—called image-based DECT [17]—in contrast to projection-based DECT; this type has very limited capabilities in terms of reducing the beam-hardening artifacts [25].

The biggest advantages of dual-source scanners are their extraordinarily high temporal resolution up to 66 ms when operating in single-energy mode [17] and their ability to independently modulate currents to reduce radiation doses and install filters in order to increase the energy separation of emitted photons [5,9,26,27]. The temporal resolution in dual-energy mode is not so extraordinary but is still high—125 ms [17,28]. Dual-source scanners can generate 40 keV–190 keV monoenergetic images (Table 1) [5].

Almost twice as much hardware, meaning a higher price, and more components are a couple of the main disadvantages. The different FOV of two detectors limits the area within which dual-energy data can be calculated. This is not a problem in case of the heart due to its central location but it limits possible usage in diagnostics of other organs in larger patients [11,29]. The FOV of a detector linked with a higher voltage powered tube is 50 cm in all generations; a second detector FOV has been expanded in the next generation but it is a limiting factor of dual-energy data (Table 1). Carefully placing the patient in the center of the scanner is crucial [15].

The shift of tubes causes a minimal delay of registration data from the same location of both lamps, which can generate misregistration artifacts [29,30]. Moreover, scatter photons that originate in one tube can reach the detector of another and generate artifacts [27,29].

### 4.2. Split Filter DECT—TwinBeam

The second type of DECT developed by Siemens Healthcare, Erlangen, Germany, uses sets of X-ray tube filters to split the beam in half in the *Z*-axis. The gold filter eliminates high-energy photons from the beam generated with 120 kV kVp, while a zinc filter absorbs low-energy photons [31]. It is a cheaper solution then dual-source scanners because the only added hardware to standard scanner is sets of filters [24]. However, due to big delays in two energy registration data—the time of tube rotation with pitch = 1—this type of scanner has limited application in heart examination, which is confirmed by a lack of any publication in this field concerning this type of scanner.

### 4.3. Rapid-kVp-Switching DECT

A dual-energy solution developed by General Electric Healthcare, Waukesha, WI, USA, uses two unique elements: an X-ray tube capable of switching kVp between 80 and 140 kV [11,32] and an ultra-fast registering detector based on gemstones with shorter afterglows than traditional material used for their construction [30,32,33]. During the acquisition, the kVp of the tube changes every 0.25 ms between 80 and 140 kV, which allows one to obtain two datasets from almost same point. This allows one to use projection-based methods in reconstructing monoenergetic images [18]. In order to minimize misregistration artifacts, the speed of tube rotation is decreased [17]. The lower the voltage, the lower the number of generated photons with this same current. In order to overcome this limitation, 66% of the cycle lamp operates with 80 kV [17,26].

Imaging the entire FOV [9,18] in dual energy from almost the same location of the tube almost completely eliminates misregistration artifacts and errors in the calculation of monoenergetic images [29,30]. Very rapid changes to kVp makes it impossible to use filters to increase energy separation between low- and high-energy photons [11,30]. For that same reason, current modulation cannot be applied to reduce the radiation dose [5]. In order to use projection-based reconstruction methods, the rotation speed has to be limited because the tube has to be in the same spot or very close to the same point, which limits the temporal resolution of scanner. Moreover, fixed settings of 80 kVp and 140 kVp limit the possibility of performing the examination in obese patients due to low-energy photon starvation [15].

### 4.4. Multilayer Detector CT

The only commercially available detector-based dual-energy CT scanner was created by Philips Healthcare, Best, Netherlands, who designed a dual-layer detector sometimes called a “sandwich detector”. It consists of two layers: an inner layer that registers lower-energy photons and is transparent for high-energy ones, and an external layer that registers them [11,13]. A standard X-ray tube is used in this scanner. It is truly a projection-based DECT, which has huge advantages due to a greater possibility of artifact reduction. Similarly, as in the rapid-kVp-switching DECT, dual-energy data are available for the entire FOV, but in contrast, there is no need to reduce the rotation speed, so temporal resolution is not compromised. Working with 120 kV kVp, these scanners work as dual-energy scanners without any further modification, which improves workflow because personnel do not have to decide if a specific examination has to be in the DECT mode, like in other types of DECT. Moreover, it does not carry the penalty of an extra radiation dose or the loss of the temporal resolution of a scanner [13].

It is possible that a few low-energy photons would not be absorbed by the inner layer of the detector and reach and interact with the external one. The opposite scenario is also possible. Both would result in an artifact and the miscalculation of the error in calculating monoenergetic images.

### 4.5. Sequence DECT

The simplest method is to scan the area of examination twice with different kVp. This solution was adopted by Toshiba Medical Systems, Tochigi, Japan in their Aqulinon CT scanners, which change kVp after a full gantry rotation. This does not require any specific hardware modification, just dedicated software. Due to completely independent scanning in both cycles, techniques of dose reduction are available and obtain similar signal-to-noise ratios, which improve the quality and accuracy of monoenergetic images. Both scans from the same spot are delayed by the time of tube rotation (the minimum rotation time for Aqulinon is 0.27 s) which increases the likelihood of motion artifacts and differences in contrast concentration [2].

## 5. DECT Implementation Heart Imaging

### 5.1. Coronary Artery Assessment

**Figure 2:**
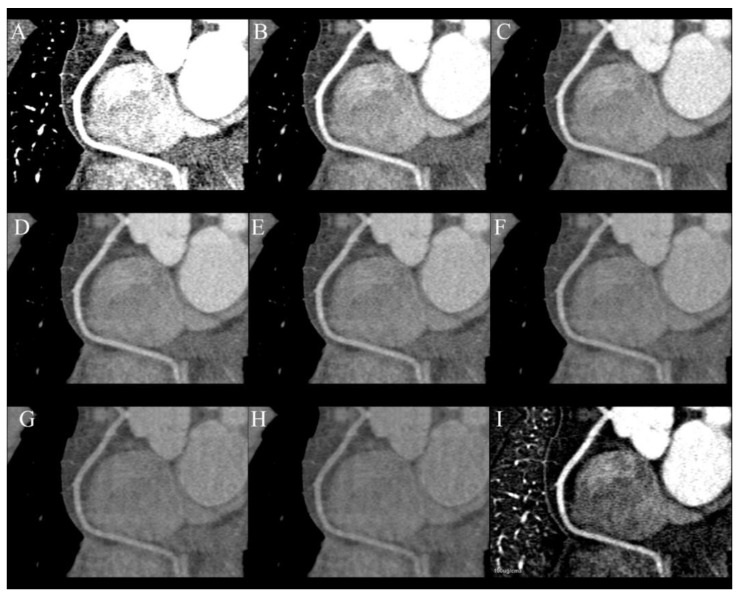
Series of curved MPR of RCA at different energy VMIs: (**A**)—40 keV, (**B**)—50 keV, (**C**)—60 keV, (**D**)—70 keV, (**E**)—80 keV, (**F**)—100 keV, (**G**)—120 keV, (**H**)—140 keV. The best contrast to noise ratio is at 60–70 keV images (**C**,**D**) Lower energies have higher iodine density but also much higher noise. Higher-energy VMIs (**F**–**H**) are less useful due to low contrast density. (**I**)—curved MPR reconstructed from iodine(water) map can also be used to assess lumen of RCA.

The primary goal of heart CT is the assessment of coronary arteries and improving quality and diagnostic possibilities is the main reason for performing this examination in dual-energy mode (Figure 2A–I). This is possible by reducing the number of blooming artifacts from stents and calcifications. Monoenergetic images, material specific reconstruction and effective Z-number imaging are helpful in reaching that goal [13,34]. The blooming artifacts that originate from stents and calcified plaques are a reason for the overestimation of the degree of stenosis. They can be reduced by VMIs of high energy, e.g., 110 keV, which has been proven to highly reduce artifacts from hyperdense metallic structures, but simultaneously, they are less sensitive to iodine. For that reason, it is essential to assess the lumen of coronary arteries using multiple VMIs. Calcium subtraction is other method of increasing the accuracy of heavily calcified coronary arteries [24,34,35].

The same phenomenon makes the assessment of stents in lumen challenging. This problem was researched in detail by Hickethier et al. [36]. They reported that the amount and severity of blooming and beam-hardening artifacts depends on the stent’s material and its structure. The VMIs are significantly more effective in visualizing stents in lumen compared to standard polychromatic images as they reduce blooming artifacts, decrease noise and increase the contrast of images. VMI’s capabilities depend on the metal the stent is made of, e.g., stainless steel artifacts can be almost completely eliminated, while tantalum are just slightly reduced [36]. However, the research of Hickethier et al. did not take differences in stent structure into account, such as strut thickness, which is also related to a number of artifacts.

The degree of stenosis caused by soft plaques can be better assessed by using low-energy VMIs, e.g., 50 keV, which increase CNR and allows one to use a lower volume of contrast media [4].

**Figure 3:**
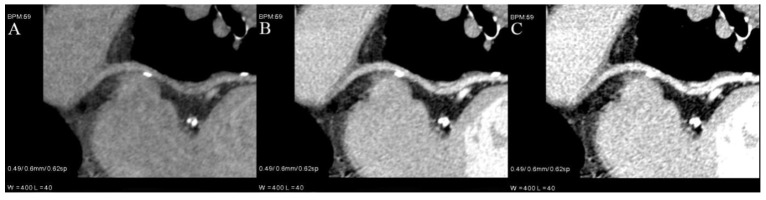
Curved VMI reconstruction of coronary graft of poorly enhanced CTA. Three reconstructions with different energies ((**A**)—80 kev, (**B**)—60 keV, (**C**)—50 keV) and the same window and level settings. Contrast density is rising with lower energies, but also noise and artifacts from metal clips around graft.

Moreover, they can be used to salvage examinations with suboptimal vessel enhancement [9,13] (Figure 3) or to asses pulmonary and coronary arteries in a single examination without an extra dose of contrast—making each coronary CTA rule out examination.

**Figure 4:**
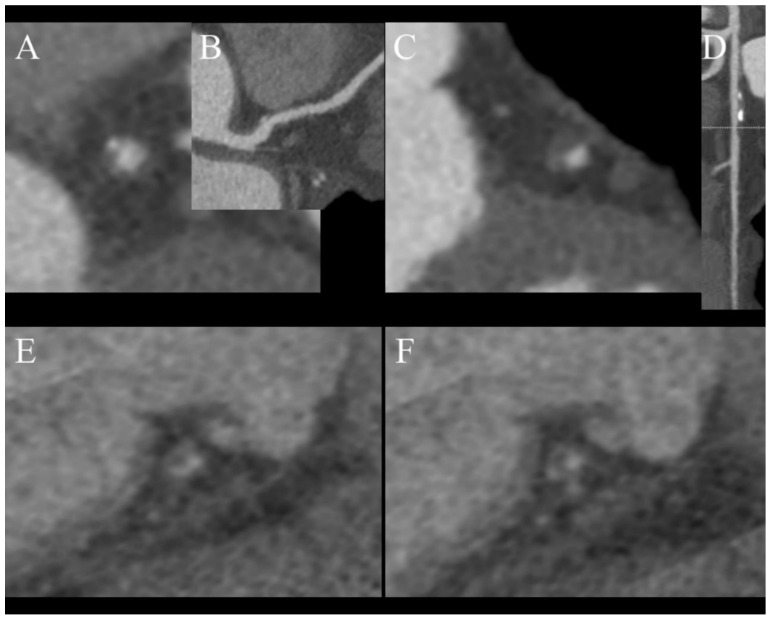
Features of venerable plaques. (**A**,**B**)—spotty calcification—calcification smaller than 3 mm and less than 150% lumen diameter (**A**)—short axis view, (**B**)—curved MPR of the vessel; (**C**,**D**)—positive wall remodeling—outer diameter of involved section of the vessel at least 110% of non-involved part of vessel; (**E**,**F**)—both plaques are noncalcified and low density (<30 HU) corresponding to cholesterol reach core, (**F**)—napkin ring sign—hyperdense outer part of noncalcified plaque.

Despite the constant development of CT scanners, invasive coronarography still has much better spatial and temporal resolutions then any CT scanner, but it cannot provide any information about plaque composition. These data are only obtainable by performing intravascular ultrasound (IVUS), which is not widely available. This information is very important because it is proven that plaques with tiny fibrous cups or a large necrotic core are prone to rupture and cause myocardial infarction, which makes them very dangerous. SECT can provide limited information about plaque composition by evaluating its density. DECT can offer much more than just plaque density, by analyzing its atomic number [37,38,39]. Several studies have proved that is possible to assess the composition of soft plaque using DECT and more accurately detect venerable ones, which can lead to more intensive and potentially beneficial treatment for patients [37,40,41]. There are some characteristic features of venerable plaques in CT:

1. Spotty calcification (Figure 4A,B);
2. Positive remodeling (Figure 4C,D);
3. Low density core (Figure 4E,F);
4. Napkin ring sign (Figure 4F).

A study by Nakajima et al. determined that using a value of 9.3 as the effective atomic number has 90% sensitivity in distinguishing soft and fibrous plaques, while density with a cutoff value of 55 HU only has 62% sensitivity. A limitation of this study is that it had a small population of just 18 patients [42]. In summary, plaque characterization in DECT has still not been fully researched and requires further investigation, but combining information regarding the effective atomic number with CT features of unstable plaques (Figure 3) can help to determine the nature of atherosclerotic changes in examined vessels [24]. We do not use DECT to characterize plaques in daily practice and rely on CT futures of venerability, which were mentioned earlier.

### 5.2. Contrast Volume Reduction

By using low-energy VMIs, we can significantly increase CNR and enhance the vessels in comparison to the SECT examination using the same volume of contrast media and iodine delivery rate, or we can obtain a comparable quality of image that is obtained with a lower volume of contrast media. Several papers present examinations performed with less than 50% of standard volume of contrast without a loss of quality, which requires some modification in contrast delivery protocol [7,43,44,45]. Reduced contrast volume is especially beneficial for patients with impaired renal function, due to dose-related contrast-induced nephrotoxicity. However, this does not reduce the risk of allergic reactions which are not dose related [34,45]. The increased sensitivity to iodine of low-energy VMIs allows one to asses smaller or poorly enhanced vessels [7,9] (Figure 3). It is important to note that the same iodine concentrations may have slightly different Hounsfield unite values in the same VMI produced by different types of scanners [46]—it is very important to know the type of scanner that is installed in one’s institution.

### 5.3. Radiation Dose Reduction

**Figure 5:**
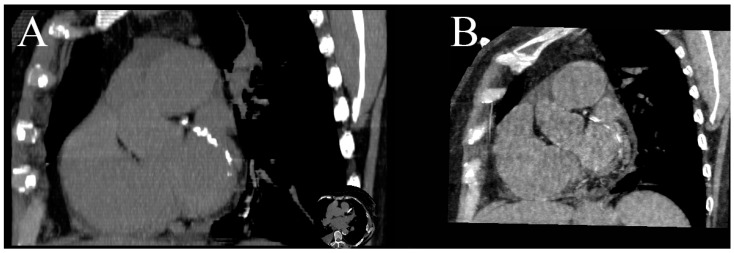
MPR MIP image of true unenhanced image (**A**) and water (iodine) map (**B**) showing calcifications in the LCx. The volume of calculated calcifications was 85 mL and 88 mL, respectively. Similar results, showing high correlation between calcium volume calculated from TUN and VUIs, were reported by [24].

Coronary CTA originally had one the highest doses of radiation of all CT examinations; thanks to the development of prospective ECG-gating, current modulation and iterative reconstruction algorithms, it has been radically decreased, even to below 1 mSv [10]. It can be further reduced by omitting the noncontrast phase and using DECT’s capabilities of creating virtual unenhanced images (VUIs) [13,24]. Many phantoms and human-based experiments have proved that there is a strong correlation between the Agatston score calculated from the real unenhanced images and VUIs. However, none of the vendors that provide DECT scanners have FDA- or EU-approved Agatston scoring software for use with DECT contrast images [4,10,14,24,47]. In order to use calcium scoring from VUIs in routine clinical practice, precise software has to be modified, because it uses a threshold of 130 HU to extract calcium, whereas water (iodine) maps are coded in element concentrations, making automatic extraction impossible (Figure 5). Most vendors also offer a method of obtaining VUIs coded with Hounsfield units, but this method tends to misclassify small calcification as iodine and extract them as well.

The greatest dose reduction can be achieved using third-generation dual-source scanners capable of performing coronary CTA with pitch = 3 and a submillisievert radiation dose, but this mode can be used only in patients with low heart rates [10].

### 5.4. Heart Perfusion

Coronary CTA performed with SECT can only assess the anatomy of coronary arteries and the degree of stenosis, but with almost no information on perfusion, only vast perfusion defects can be spotted as hypodense areas of myocardium. DECT CTA can be used to calculate the concentration of iodine in myocardium distally to stenosis and assess the significance of stenosis. The ability to simultaneously evaluate the morphology of stenosis and its hemodynamic significance is not available to any other method of heart imaging [4,34,48]. However, it is a static evaluation depicting the contrast in time of CTA scanning, which is usually less than 1 s. In contrast to classic perfusion CT, it does not produce information about blood flow over time.

Using a DECT scanner can reduce the beam-hardening artefact originating from concentrated contrast in SVC and the right chambers, which can mimic hypoperfused areas of the myocardium and increase the accuracy of first-pass perfusion compared to SECT [9,34,48,49].

Dynamic examination is much more accurate than static and gives the opportunity to perform a quantitative assessment of myocardial perfusion but with a higher radiation dose. Some centers perform coronary CTA, which is also used as static rest perfusion and dynamic examination under pharmacological stress to assess the influence of detected changes on blood flow during one patient visit to a CT lab [48,50,51]. his protocol offers the most complex assessment of coronary arteries and can be used to roll out any significant stenosis, but comes with the price of a relatively high radiation dose [52]. Both dynamic and static DECT heart perfusion have been evaluated against SPECT, MRI, PET, FFR and FFR_CT_ with very good correlations in several studies, which demonstrates that dynamic examination is more sensitive and specific then static [53,54,55,56,57]. A recent study published by Ruiz-Muñoz et al. demonstrated the superiority of dual-energy perfusion over single-energy with better sensitivity, specificity, negative and positive predictive value in detecting significant stenosis with SPECT and invasive coronarography used as standard [58]. Similar conclusions of better accuracy of static dual-energy perfusion over single-energy were stated by Assen et al., but both methods were inferior to dynamic perfusion [59]. The large multi-center clinical trial DECIDE-Gold was launched in 2014 in order to determine DECT perfusion in detecting significant coronary artery disease, but its results have not been published yet [60].

### 5.5. Myocarditis and Fibrosis

**Figure 6:**
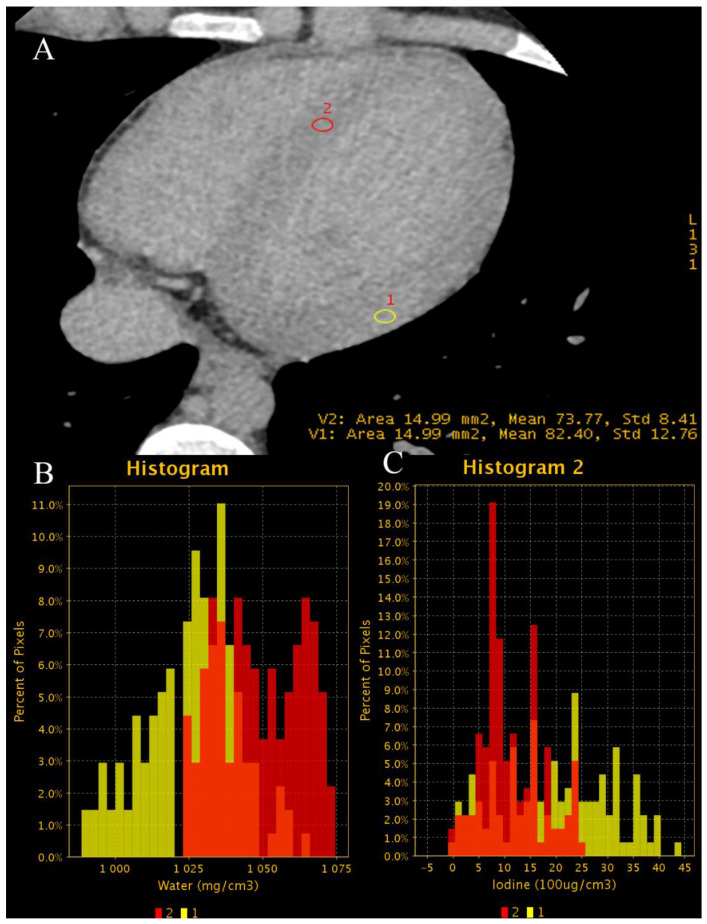
At 70 keV VMI, the subepicardial region of lateral wall of increased density is visible in delayed phase (**A**); it is caused by increased iodine uptake and reduced water concentration (**B**,**C**).

The modality of choice in diagnostic of myocarditis and myocardial fibrosis is magnetic resonance with gadolinium contrast injection and the assessment of late gadolinium enhancement (LGE); however, some patients have contraindications for MR examination. For this population, heart CT with delayed phase is an alternative method, especially when performed in the dual-energy mode. Inflammatory processes locally disturb the function of ion pumps and result in the leaking of gadolinium or iodine from vassals into peripheral tissues, resulting in an enhancement better seen in late phases due to the trapping of contrast in the site of inflammation and washout from normal muscle [50]. Low-energy VMIs and iodine(water) maps can clearly show regions of even weak enhancement and can be used to measure iodine concentration in the case of doubt [61] (Figure 6A–C). It was proved by Ohta et al. that due to similar the pharmacokinetic properties of gadolinium and iodine-based contrast, DECT can be used in differentiating ischemic and non-ischemic cardiomyopathies in patients with heart failure. They report the better concordance of iodine density maps with LGE in CMR studies than low keV VMIs [62]. A similar conclusion was provided by the study of Matsuda et al., who confirmed that late iodine enchantment can be used as a substitute of LGE in diagnostic infarction [63]. Adding late-phase acquired in the dual-energy mode to perfusion examination can increase the sensitivity and specificity in detecting areas of infarction [59].

### 5.6. Differentiating Thrombus, Tumor and Artifacts

**Figure 7:**
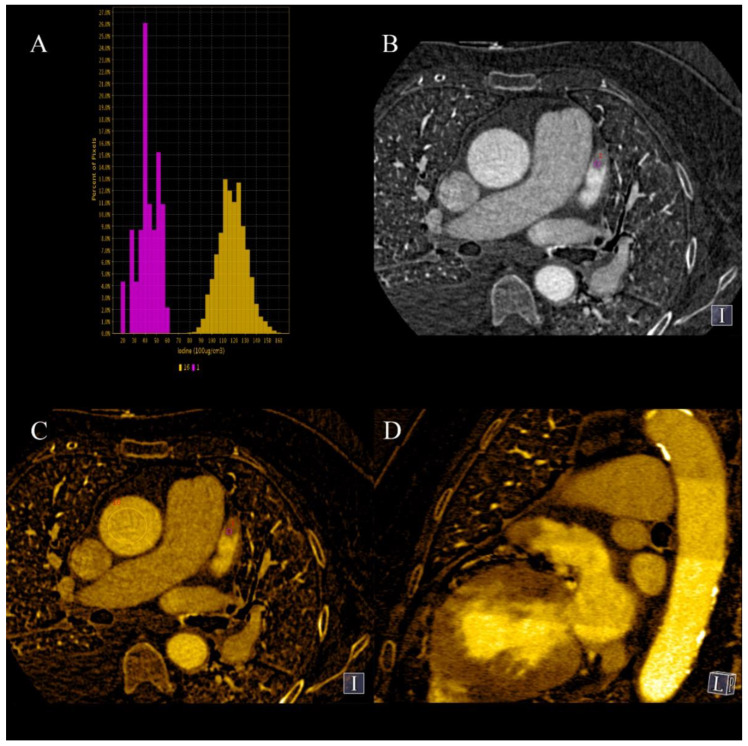
(**A**–**D**). Case of patient with chronic atrial fibrillation after 3 unsuccessful ablations, currently admitted due to chest pain. Coronary CTA was performed to roll out coronary artery stenosis. Differentiating thrombus and filling defect of LAA using iodine concentration is much more specific and sensitive than the use of Hounsfield unities ratio, as proven by Hur et al. [64]. (**A**)—Histogram of iodine concentration in ROIs; (**B**)—map iodine(water) shows lower concentration of iodine in LAA of about 4.1 mg/mL, meeting the criteria for circulatory stasis by Hur et al.; (**C**,**D**)–70 keV VMI with color overlay of iodine(water) map. (**D**)–in log axis of LAA. In the lower right corner of sub-image B-C-D information about viewpoint.

There are three causes of contrast filling defects of heart chambers in coronary CTA: a thrombus, a tumor or a blood flow artefact. The first two require further diagnostic investigation due to different treatments. The most common location of contrast filling defects in patients with atrial fibrillation is the left atrial appendage (LAA); it is also the most common location of intracardiac thrombus. Definite differentiation is possible by performing an additional scan in the venous phase but has the disadvantage of additional radiation dose; definite differentiation can also be achieved by transesophageal echocardiography (TEE). DECT, by using low-energy VMIs or iodine(water) maps can detect even minimal concentrations of iodine in LAA and exclude the presence of thrombus. The measurements of iodine concentration are more accurate than the density of LAA, as it is carried out in SECT (Figure 7A–D) [9,64], and as proven by Hur et al., using 1.74 mg I/mL as the cutoff value for thrombus has 100% specificity. The third cause of the filling defect is the presence of a tumor in the chamber of the heart. As proven by Hong et al., it is possible to differentiate tumors and thrombus using dual-energy coronary CTA by measuring the iodine concentration [65,66].

### 5.7. Diagnosis of Implant-Related Pathologies

**Figure 8:**
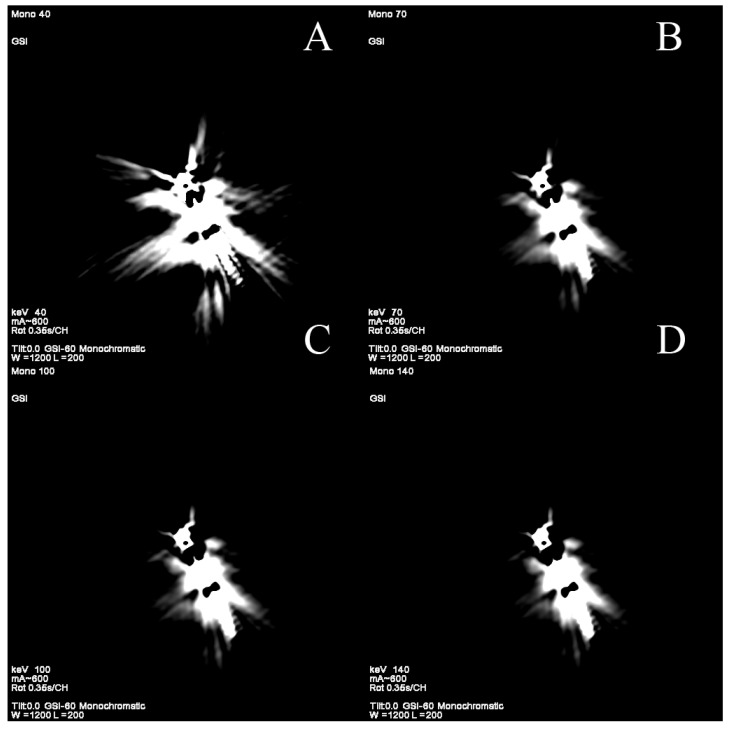
(**A**–**D**). Reduction in artifacts originating from pacemaker electrode using high-energy VMI (**C**,**D**). Phantom study. GSI, gemstone spectral imaging.

There is a growing population of patients with implemented intracardiac devices such as artificial valves and electrodes of pacemakers, which can become the source of complications—the electrodes can break or perforate the heart wall. Besides, every foreign material can be colonized by pathogens and became the source of endocarditis. Visualizing vegetation on the surface of electrodes or parts of artificial valves can be hard due to beam hardening, blooming and photon starvation artifacts. These artifacts can completely obscure small vegetations, which makes it had to detect them using SECT [13,67]. It has been proven that DECT can significantly reduce metal-related artifacts originating from artificial valves or electrodes (Figure 8A–D) [68].

### 5.8. Reduction of Metal-Related Artifacts

Very dense materials such as metal clips, electrodes or stents and massive calcifications cause artifacts due to the much higher absorption of X-ray photons than surrounding tissues. These phenomena, in combination with how CT scanners reconstruct images, are reasons why such structures are the source of many types of artifacts such as beam hardening, blooming and photon starvation [69,70,71].

The beam hardening artifact occurs when a polychromatic X-ray beam passes through a high-density structure, which absorbs disproportionally more low-energy photons than high-energy ones. This disproportion generates hyper- and hypodense streaks on reconstructed images [5,69,72]. VMIs are much more resistant to these artifacts because they simulate images obtained with photons of single energy—is it not possible to harden that beam, only to attenuate it.

Photon starvation artifacts are hypodense areas with increased noise around massive and dense structures due to the absorption of almost all photons by them. We can see them between hip prostheses or superior thoracic apertures. They are best seen in coronal MPR.

Blooming artifacts are induced by the partial volume effect, which is related to the method of how scanners measure density and reconstruct images. As a result, hyperdense objects appear to be bigger than they really are, which in the case of stents or calcifications, causes narrowing of the lumen. Stehli et al. proved that VMIs of 80 keV and more are superior to SECT in reducing this type of artifact [73].

Stent- and surgical clip-related artifacts are problems in the assessment of arterial lumen during coronary CTA, due to the abovementioned types of artifacts, which makes them appear bigger and the lumen smaller, which leads to the overestimation of stenosis and unnecessary invasive coronarography. VMIs and material-specific images, especially iodine(water), are useful in reducing artifacts [6,8,74], but they cannot eliminate them [73]. Moreover, the latest CT scanners, both DECT and SECT, are equipped with metal reduction algorithms that can be additionally applied to increased image quality, e.g., MARS in GE scanners [75], O-MAR in Philips Healthcare, Best, Netherlands or iMAR in Siemens Healthcare, Erlangen, Germany [20].

The severity of artifacts and DECT’s ability to reduce them is related to stents’ structures and their composition. Nitionol structures create few artifacts, which are significantly reduced, whereas tantalum structures are sources of severe artifacts that are almost resistant to reduction using VMIs. Stent diameter is also a very important factor that influences the severity of artifacts [36,74].

## 6. Incidental Extracardiac Findings

Quite often in the scanning area of coronary CTA, there are some important pathologies, such as enlarged lymph nodes, pulmonary nodules, liver changes or nodules in adrenal glands. It has been proved that DECT can differentiate metastatic lymph nodes from inflammatory [12,76,77], malignant nodules and benign nodules in lungs [12,76,78,79,80] and adrenal glands [76].

**Figure 9:**
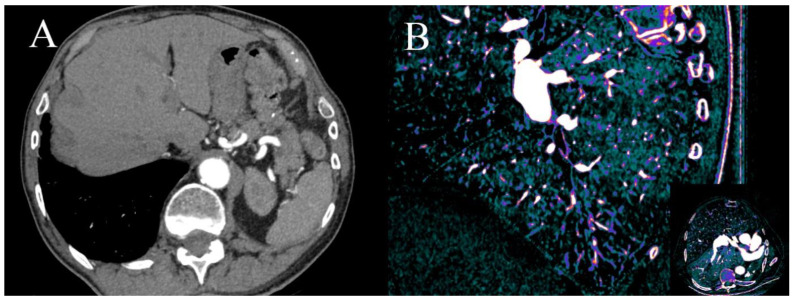
Dual-energy coronary CTA performed due to worsening of dyspnea and suspected CAD. There is small clot in peripheral artery in segment 9 of right lung which can be easily misted in axial VMI at 70 keV (**A**). There is a large V-shape aerial of hypoperfusion visible in sagittal reformat on iodine(water) map in segment 9 (**B**); similar aerials were discovered in segments: 4R and 5R. Finally, diagnosis of chronic peripheral pulmonary embolism was made.

DECT pulmonary CTA is the most sensitive method of detecting pulmonary embolism, but coronary CTA performs a few seconds after the contrast travels from the pulmonary circulation into systemic circulation. Its concentration is too low in pulmonary arteries to use them using SECT, but low-energy VMIs and iodine(water) maps allow the assessment of pulmonary circulation (Figure 9) [12,16,81,82].

## 7. Impact of DECT on Workflow in Radiology Department

Dual-energy CT has greater diagnostic capabilities then SECT. Each type of scanner has its own unique advantages, disadvantages and limitations. When planning to purchase such a device, one should take into consideration what kind of examinations will mainly be performed on that scanner. If other types of examinations will also be performed, the main areas of diagnostic and scientific interest of the specific department have to be considered.

Besides the technical ability to perform dual-energy examination, the knowledge of how to interpret them is even more important. Training in the interpretation of dual-energy examinations by radiologists is time-consuming, and to be cost-effective, close cooperation of the radiologist and radiographer is required.

In every type of DECT, except the multi-layer detector, it is necessary to plan specific examinations to be dual energy, which requires some work planning and patient selection. It is possible to perform every examination as a dual energy examination, but in some cases there will be no useful information and patients will be exposed to an additional dose of radiation. Every institution working with a DECT scanner has to develop its own way of organizing work with this type of machine. In our department, we select patients for DECT examination if the referral suggests pathologies that can be better assessed in that mode or if previous examinations were inconclusive.

Due to their complexity, DECT scanners are more expensive than SECT ones, so the installation of them should be thoroughly thought out.

## 8. Limitations of DECT

Dual-energy CT has many advantages over SECT, so why it is not wildly used? The main reason is probably the lack of radiologists’ and hospital ménages’ knowledge about its capabilities. Moreover, complicated, often unintuitive and expensive software necessary to fully use the potential of this technology is required. Dual-energy scanners are about 25% more expensive to buy and operate than single-energy devices of similar class due to the highly complex elements produced exclusively for them, which increases their cost due to their small quantity. Furthermore, DECT, as with every imaging modality, has some limitations strongly connected with the type of scanner. The rapid-kVp-switching DECT is prone to a motion artifact due to inferior temporal resolution, but they offer good energy separation and projection-based VMI reconstruction. Twin-beam, dual-source and sequential DECT scanners have better temporal resolution but come with the price of delayed registration of a second energy dataset and the possible miscalculation of VMIs. Sandwich detector scanners allow for the simultaneous registration of both energy datasets but are at risk of artifacts due to the misregistration of photons by the wrong layer of the detector.

## 9. The Future of Heart DECT

The current applications of DECT in heart diagnostics are presented in Table 3, and the most important studies comparing DECT with other modalities are presented in Table 4, which determines their sensitivity and specificity. Researchers are continuously looking for new applications for DECT in many fields, including the heart. There are several papers that describe the ability of DECT to estimate extracellular volume (ECV), which is helpful in diagnostics of cardiomyopathies. Until recently, only CMR was able to measure ECV. There are some discrepancies in the formulas used to calculate ECV depending on the type (image- or projection-based) of scanner [83,84]. The accuracy of this method has been proved in comparison with CMR and histological sampling [83,85]. DECT is the only one-stop imaging modality that allows one to assess ECV and the coronary arteries simultaneously, as well as simultaneously assessing perfusion, coronary arteries and plaque to predict their stability. This wide range of information that can be obtained during one examination is beyond the reach of invasive coronarography. It has been proved in many trails, e.g., the SCOT-HEART trail, that using CTA is cost-effective in the care of patients with stable chest pain and it reduces the risk of cardiac death [86]. Adding DECT capabilities may only improve the detection rate of hemodynamically significant stenosis.

## Figures and Tables

**Table 1:** Main differences between types of DECT and technical details of the latest models of each type. All types except dual-source offer 50 cm FOV.

Manufacturer	GEHealthcare, Waukesha, WI, USA [17,18]	Philips Healthcare, Best, Netherlands [13,19]	Siemens Healthcare, Erlangen, German [20]	Siemens Healthcare, Erlangen, Germany [21]	Cannon Medical Systems, Tochigi, Japan [22,23]
Latest models	Revolution	IQon Spectral CT	SOMATOM Force (3rd generation)	SOMATOM Definition Edge	The Aquilion ONE VISION
Type name of DECT	Rapid kVp switching (GSI)	Multilayer detector (“sandwich detector”)	Dual Source Dual Energy	TwinBeam Dual Energy	kV switching between gantry rotation
	Source-based technology	Detector-based technology	Source-based technology	Source-based technology	Source-based technology
VMI generation technique [24]	Projection-based	Projection-based	Image-based	Image-based	Image-based
FOV in DECT (cm)	50	50	35.6 for 3rd generation (more details in Table 2)	50	50
Filters in DE mode	No	No	Yes, tin filter for higher potential tube	Yes, split gold and tin filter	no
Z-cover (mm)	160	40	115.2	64	160
Rows	256	64 rows 256 slices	2 × 192	128	320
Slice thickness (mm)	0.625	0.5	0.6	0.5	0.5
Automatic Exposure Control	No	Yes	Independent for both lamps	no	Yes, mA modulation independent for each rotation [23]
Energy levels’ range of monoenergetic reconstructions	40–140 keV	40–200 keV	40–190 keV	40–190 keV	35–135 keV
Minimal rotation time	0.5 s in dual-energy mode	0.27 s	0.25 s	0.28 s	0.275 s
Temporal resolution	250 ms	135 ms	66 ms	142 ms	137.5 ms [23]
Spatial resolution	0.23 mm	N/A	0.3 mm	0.3 mm	0.17 mm
Dual-energy data registration offset (ms)	0.25 ms	None	66 ms	One gantry rotation with pitch 1	One gantry rotation

DECT, dual-energy CT; VMI, Virtual Monoenergetic Image; FOV, field of view, DE, dual energy.

**Table 2:** The latest generation of dual-source scanners offers best temporal resolution with greater dual-energy FOV and longer *Z*-axis coverage. The energy separation was also increased which reduce energy overlapping [17].

Generation		First	Second	Third
Model		SOMATOM Definition	SOMATON Definition Flash	SOMATOM Force
FOV of lower voltage X-ray tube—dual-energy FOV		26 cm	33 cm	35.5 cm
Detector rows		2 × 64	2 × 128	2 × 192
Slice thickness		0.6	0.6	0.6
Temporal resolution (ms)		83	75	66
Dual-energy data registration offset (ms) [17]		83	75	66
kVp	Highvoltage tube (kVp) [17]	140	140 ± Sn filter	140 Sn filter 150 + Sn filter
	Low voltage tube (kVp)	80	80 100	70, 80, 90, 100

**Table 3:** Summary of DECT advantages and its current uses in clinical situations.

Technique	Benefits	Clinical Application
Low-energy virtual monoenergetic images	Higher sensitivity for iodine.	Salvage of suboptimal contrast study. Reduction in contrast dose. Every contrast CT can have CT angiography quality. Detection of pulmonary embolism during coronary CTA.
High-energy virtual monoenergetic images	Reduction in beam-hardening and metal-related artifacts. Reduction in calcium blooming artifacts.	Better visualization of stents lumen. Better visualization of heavily calcified vessels. Reduction in artifacts from IDC electrodes, valve prosthesis.
68–70 keV virtual monoenergetic images	Best CNR virtual monoenergetic images for angiographic studies.	Increased quality of any angiographic CT.
Iodine map	Better sensitivity for iodine.	Myocardial perfusion defects. Better detection of late contrast enhancement in inflammation. Differentiating thrombus from tumor or contrast flow artifacts. Detection of pulmonary embolism during coronary CTA.
Virtual unenhanced images	Reduction in radiation dose. Reduction in time of examination.	Calcium scoring performed from angiographic phase. Characteristic of incidental findings in angiographic phase, e.g., adrenal glands tumor.
Material decomposition	Identification of tissue composition.	Differentiation of hyperdense structures. Better separation of iodine from calcium. Plaque characterization.

IDC, implanted cardiac device; CTA, computed tomography angiography; CNR, contrast noise ratio.

**Table 4:** Summary of sensitivity, specificity, positive predicting value (PPV), negative predictive value (NPV) and significant details of citated original study comparing DECT with other modalities. n/a—not available.

Author	Type of Scanner	Number of Analyzed Patients	Date of Publication	Application	Modality Used as Reference Standard	Sensitivity	Specificity	PPV	NPV
Yunaga et al. [35]	rapid-kVp-switching DECT	67	2017	Assessment of heavily calcified segments of coronary arteries using VMI	Invasive coronarography	91.30%	70.60%	55.80%	95.20%
Yunaga et al. [35]	rapid-kVp-switching DECT	67	2017	Assessment of heavily calcified segments of coronary arteries using material density image	Invasive coronarography	88.40%	88.20%	75.30%	94.90%
Obaid et al. [37]	DSCT	20	2014	Plaque composition	VH-IVUS and histopathology	64%	98%	95%	83%
Nakajima et al. [42]	rapid-kVp-switching DECT	18	2017	Using effective atomic number (EAN) to classify non-calcified coronary plaques	IVUS	90% For cutoff EAN = 9.3	87% For cutoff EAN = 9.3	n/a	n/a
Delgado et al. [28]	DSCT	56	2013	Adenosine stress static myocardial perfusion	MRI	76%	99%	89%	98%
Delgado et al. [28]	DSCT	56	2013	Ischemia detection—late enhancement	MRI	64%	99%	82%	99%
Ko et al. [50]	DSCT	41	2010	Adenosine stress perfusion	MRI	89%	78%	n/a	n/a
Ko et al. [52]	DSCT	45	2011	Dual-energy, static, stress perfusion + CTA in detecting significant stenosis	Invasive coronarography	93.20%	85.50%	88.30%	91.40%
Weininger et al. [55]	DSCT	20	2010	Dual-energy dynamic perfusion + delayed enhancement in detection perfusion defects	MRI	93%	99%	92%	96%
Weininger et al. [55]	DSCT	20	2010	Dual-energy dynamic perfusion + delayed enhancement in detection perfusion defects	SPECT	94%	98%	92%	94%
Ruiz-Muñoz et al. [58]	rapid-kVp-switching DECT	84	2021	static stress CTP dual-energy vs. single-energy	SPECT + Invasive coronarography	87%	99%	93%	98%
Bouleti et al. [61]	rapid-kVp-switching DECT	20	2017	Use of delayed enhancement in detection of myocardial infarction	MRI	100%	99%	94%	95%
Yasutoshi et al. [62]	rapid-kVp-switching DECT	44	2018	Usage of delayed enhancement in myocardial scare classification	MRI	92%	98%	n/a	n/a
Matsuda et al. [63]	DSCT	19	2015	Assessment of late enhancement with denoise filter in assessment of myocardial scare	MRI	81%	96%	81%	96%
Hur et al. [64]	rapid-kVp-switching DECT	32	2012	Differentiation between thrombus and circulatory stasis in LAA	TEE	97%	100%	100%	97%
Hong et al. [65]	rapid-kVp-switching DECT	55	2014	Differentiation between thrombus and myxoma	TTE	94%	100%	n/a	n/a
Hong et al. [66]	rapid-kVp-switching DECT	28	2018	Differentiation between thrombus and tumor	MRI	66.70%	79%	n/a	n/a
Yang et al. [77]	rapid-kVp-switching DECT	84	2017	Differentiating metastatic and non-metastatic lymph nodes in NSCL	Histopathology	88.20%	88.40%	85.80%	90.40%
Zhang et al. [78]	rapid-kVp-switching DECT	63	2016	Differentiation between malignant and benign solitary pulmonary nodules	Histopathology	93.80%	85.70%	n/a	n/a
Ruzsics et al. [87]	DSCT	36	2009	Assessment of coronary artery stenosis and of the myocardial blood supply	SPECT + Invasive coronarography	92%	93%	n/a	n/a

MRI, magnetic resonance imaging; NSCL, non small cell lung carcinoma; SPECT, single photon emission computed tomography; TEE, transesophageal echocardiogram; TTE, transthorakale echokardiographie; DSCT, dual-source computed tomography; VH-IVUS, virtual histology intravascular ultrasound; CTP, computed tomography perfusion; CTA, computed tomography angiography; LAA, left atrial appendage.
